# Seek and you shall find: *Yersinia enterocolitica* in Ireland’s drinking water

**DOI:** 10.1007/s11845-024-03641-5

**Published:** 2024-02-21

**Authors:** James Powell, Maureen Daly, Nuala H. O’Connell, Colum P. Dunne

**Affiliations:** 1 Department of Microbiology, University Hospital Limerick, Limerick, Ireland; 2 School of Medicine and Centre for Interventions in Infection, Inflammation, and Immunity (4I), University of Limerick, Limerick, Ireland; 3 School of Medicine, University of Limerick, Limerick, Ireland

**Keywords:** Drinking water, Ireland, *Yersinia*, *Yersinia enterocolitica*, *Yersinia proxima*, Yersiniosis

## Abstract

**Introduction:**

Three *Yersinia* species were identified from samples of drinking water from diverse geographic regions of Ireland. Conventional commercial biochemical identification systems classified them as *Yersinia enterocolitica*. Since this organism is the most common cause of bacterial gastroenteritis in some countries, further investigation was warranted. The aim of the study was to provide a microbial characterisation of three Yersinia species, to determine their pathogenicity, and to review the incidence rate of *Yersinia enterocolitica* detection in our region.

**Methods:**

Organism identification was performed using conventional commercial diagnostic systems MALDI-TOF, API 20E, API 50CHE, TREK Sensititre GNID and Vitek 2 GN, and whole genome sequencing (WGS) was performed. Historical data for detections was extracted from the lab system for 2008 to 2023.

**Results:**

All three isolates gave “good” identifications of *Yersinia enterocolitica* on conventional systems. Further analysis by WGS matched two of the isolates with recently described *Yersinia proxima*, and the third was a member of the non-pathogenic *Yersinia enterocolitica* clade 1Aa.

**Discussion:**

Our analysis of these three isolates deemed them to be *Yersinia* species not known currently to be pathogenic, but determining this necessitated the use of next-generation sequencing and advanced bioinformatics. Our work highlights the importance of having this technology available to public laboratories, either locally or in a national reference laboratory. The introduction of molecular technologies for the detection of *Yersinia* species may increase the rate of detections. Accurate identification of significant pathogens in environmental, public health and clinical microbiology laboratories is critically important for the protection of society.

**Supplementary Information:**

The online version contains supplementary material available at 10.1007/s11845-024-03641-5.

## Introduction

*Yersinia* species, along with the closely related genera *Chania*, *Ewingella*, *Rahnella*, *Rouxiella*, and *Serratia* constitute the *Yersinia-Serratia* clade of the order Enterobacterales, more recently classified as *Yersiniaceae* fam. nov. [1]. *Yersinia* species are small, non-spore-forming, Gram negative, rod-shaped bacteria. They are motile facultative anaerobes, oxidase-negative, catalase-positive glucose fermenters. There is evident confusion regarding the number of species in the genus; in the last 3 years, reports range from 11 [2, 3] to 28 [4] species, and also, 17 [5], 18 [6, 7], 19 [8] and 27 [9] species were reported in this timeframe. The best-known species are *Yersinia pestis*, the cause of plague and a potential biological weapon [10], *Yersinia enterocolitica*, and *Yersinia pseudotuberculosis* that are capable of causing yersiniosis, an illness that commonly presents as bacterial gastroenteritis but can manifest as terminal ileitis, mesenteric adenitis, septicemia or reactive arthritis [7]. *Yersinia ruckeri* is rarely implicated in human infections [11] but is a significant pathogen of fish so is of economic importance in aquaculture, particularly of salmonids. None of the other *Yersinia* species are considered pathogenic, so most of the available research to date has focused on these four species, with little information available on the other less virulent species [12].

We performed a study of antimicrobial resistance in bacterial isolates detected from drinking water samples collected from private and public water supplies around Ireland [13]. Three of the 201 isolates in the study were identified initially as *Yersinia enterocolitica*. These three isolates were reported simply as “coliform” as per the conventional operating procedures followed nationally and internationally (International Organization for Standardization (ISO) 9308–2:2012) [14]. *Yersinia enterocolitica* is not a common cause of gastroenteritis in Ireland; the national notification rate per 100,000 population in 2020 was 0.26, compared with 17.3 for Verotoxigenic *Escherichia coli* and 51.1 for *Campylobacter* [15]. Nevertheless, *Yersinia enterocolitica* is the most common cause of bacterial gastroenteritis in some countries [3], and since these isolates were detected from drinking water supplies for human consumption, further investigation was warranted.

Faeces specimens are not tested routinely for *Y. enterocolitica* in our laboratory; they are tested selectively where certain clinical details are provided on the sample request form. Specifically, these are abdominal pain, appendicitis, mesenteric lymphadenitis and terminal ileitis. The aim of this study was to provide a biochemical and molecular characterisation of the three isolates detected from drinking water, to determine their pathogenic potential and to interpret these findings in the context of their potential public health consequences. We also aim to provide an epidemiological review of *Y. enterocolitica* detection from clinical gastroenteritis diagnostic specimens in our laboratory over a 16-year period, in light of the detection of this organism from drinking water in our region.

## Methods

For background, University Hospital Limerick is a large tertiary care university teaching hospital in the Mid-West of Ireland. Much of our research to date has focussed on hospital-acquired infections including outbreaks of Multi-Drug Resistant Organisms (MDROs) [16–18] and other pathogens [19–22].

Phenotypic testing was performed on all three isolates in parallel with the type strain *Yersinia enterocolitica* (NCTC 10938). Microscopic morphological appearance was determined by Gram staining. Oxidase activity was tested with OXItest strips (Erba Lachema, Brno, Czech Republic). Catalase activity was assessed by the observation of bubble production in a 3% (v/v) hydrogen peroxide solution. Colonial appearance was determined using MacConkey Agar, Columbia Agar and *Yersinia* Selective Agar (Oxoid Ltd, Thermo Fisher Scientific, MA, USA). Biochemical characterisation was performed using the Sensititre GNID (Trek Diagnostic Systems, Thermo Fisher Scientific, MA, USA), the Vitek 2 GN Card and the API 20 E (both Biomerieux, Marcy-l’Étoile, France). The isolates were also characterised by mass spectrometry using the MALDI Biotyper, database version 11.0.0.0_9607-10833 (Bruker Corporation, Billerica, MA, USA).

Whole genome sequencing was performed by DSMZ Services (“DSMZ”), Leibniz-Institut DSMZ–Deutsche Sammlung von Mikroorganismen und Zellkulturen GmbH, Braunschweig, Germany. Genomic DNA extraction was completed using MasterPure™ Gram Positive DNA purification kits from Epicentre® Biotechnologies (Germany) according to the manufacturer’s instructions. Libraries were prepared by applying the Nextera XT DNA library preparation kit (Illumina®, USA). Samples were sequenced on a MiSeq sequencing system from Illumina, using MiSeq Reagent Kit V2. Genomes were assembled via SpaDES on short-read genome data, which were recorded in-house at DSMZ. After genome assembly, contigs were annotated via Prokka and analysed via the DSMZ in-house type strain genome server (TYGS), a free online bioinformatics platform [23]. All contigs shorter than 1000 bp were deleted since these are artificial contigs often produced by low coverage. Information on nomenclature, synonymy and associated taxonomic literature was provided by TYGS’s sister database, the List of Prokaryotic names with Standing in Nomenclature (LPSN, available at https://lpsn.dsmz.de) [24].

The retrospective review of *Y. enterocolitica* detection from faeces specimens was performed by extracting all relevant laboratory test results from 2008 to 2023 from the Laboratory Information Management System (LIMS, iLab, DXC Technologies). Specimens tested for *Y. enterocolitica* in our laboratory are cultured directly onto *Yersinia* Selective Agar (Oxoid Ltd, Thermo Fisher Scientific, MA, USA).

## Results

The three *Yersinia* isolates were from geographically diverse sampling sites, one from a public drinking water fountain in the South East of the country (“Isolate A”), one from a private supply in the East Midlands (“Isolate B”) and one from a private supply type in the Mid-West (“Isolate C”). For geographical locations of the sampling sites of these three isolates and the laboratories that they were detected in, please see Supplementary Fig. 1. All three isolates were resistant to the β-lactam amoxicillin and the β-lactam/β-lactamase inhibitor co-amoxiclav. One isolate (“Isolate A”) was also resistant to the antimicrobial chloramphenicol. The three *Yersinia* isolates comprised catalase positive, oxidase negative, Gram negative rods. Colonies on *Yersinia* Selective Agar at 24 h were moist, convex and circular, with pink-red centres surrounded by a transparent border, indistinguishable between the three isolates. The three isolates also grew well on MacConkey Agar and Colombia Agar at 28. 5 °C (± 0.5 °C), 36. 5 °C (± 0.5 °C) and 42.5 °C (± 0.5 °C). MALDI-TOF provided an identification of *Yersinia enterocolitica* for all three isolates, with log scores of greater than 2.2 (log scores above 2.0 are assigned the label “high confidence identification”). The API 20E results were identical for Isolate A, Isolate B and the type strain, with a 92.5% identification of *Yersinia enterocolitica*. Isolate C had one biochemical test difference (L-ornithine) so had the same identification (*Yersinia enterocolitica*), but at a lower score (81.5%). The API results represented a “very good identification” to the (*Yersinia*) genus. See Supplementary Table 1 for API 20E results.

Sensititre GNID results were almost identical for the three wild strains, providing an “unreliable identification” of *Yersinia intermedia*/*Yersinia enterocolitica* subsp. *enterocolitica*. The additional test indole was recommended, and when the result (negative) was added, 100% identification of *Yersinia enterocolitica* subsp. *enterocolitica* was confirmed. The results for the type strain reflected 100% identification of *Yersinia enterocolitica* subsp. *enterocolitica* without any additional tests. See Supplementary Table 2 for Sensititre GNID results.

The Vitek 2 GN identification for Isolate A was a “low discrimination” identification of *Yersinia enterocolitica/frederiksenii*, with rhamnose offered as a test to separate the two species. When rhamnose (negative) was added (result taken from the API 20E), *Yersinia enterocolitica* was the suggested identification. Isolates B and C both had “good identification” as *Yersinia enterocolitica/frederiksenii*, and when the rhamnose (negative) result was added, *Yersinia enterocolitica* was the suggested identification. The type strain had an “excellent identification” of *Yersinia enterocolitica/frederiksenii*, and when the rhamnose (negative) result was added, *Yersinia enterocolitica* was the suggested identification. The Vitek GN results can be seen in Supplementary Table 3.

Whole Genome Sequencing (WGS) was performed on the three strains. The genome of Isolate A was assembled in 41 contigs, and 2261 CoDing Sequences (CDS) were detected. Genome size was determined as 4.78 Mb. The 70% dDDH value [25] (sum of all identities found in High Scoring segment Pairs (HSPs) divided by overall HSP length) was not met for any other organism in the TYGS database for this isolate, so further molecular work was required to attain a match. The average nucleotide identity (ANI) was also calculated at 95.99% similarity with *Yersinia enterocolitica* subsp. *enterocolitica* ATCC 9610, and this value was deemed an acceptable identification level for species circumscription [26].

Isolate B was assembled in 41 contigs, and 4240 CDS were detected. The genome size was determined as 4.61 Mb. Isolate C was assembled in 33 contigs, and 4110 CDS were detected. The genome size was determined as 4.50 Mb. Both Isolate B and Isolate C were deemed to belong to *Yersinia proxima* (93.2% and 94.1% similarity, respectively) when the recommendations of a threshold value of 70% DNA-DNA similarity for the definition of bacterial species by the ad hoc committee [25] were considered.

The historical test counts for standard bacterial pathogens, test counts specifically for *Y. enterocolitica*, and positive detections of *Y. enterocolitica* from these tests are displayed in Table 1. The proportion of specimens tested for *Y. enterocolitica* has increased over the 16-year timeframe from 0.1% (39 of 33,811) in the first 5 years of the study to 1% (456 of 42,625 specimens) in the last 5 years of the study. Nevertheless, tests for this organism continue to represent a small subset of the overall tests, and the positivity rate reflects this: Five positive patients were detected over the 16-year timeframe, see Table 2 for patient details.

**Table 1:** Total faeces specimen counts, specimens tested for *Yersinia enterocolitica*, and culture-positive specimens (2008 to 2023)

	Faeces specimens	*Yersinia* culture	*Y. enterocolitica* isolated
2008	6918	5	1
2009	6448	9	1
2010	6426	11	0
2011	6959	10	0
2012	7060	4	0
2013	6950	28	0
2014	7816	56	0
2015	8083	80	0
2016	8804	76	1
2017	8267	71	0
2018	8761	62	0
2019	8864	33	0
2020	6789	64	0
2021	7513	108	1
2022	9146	124	0
2023	10313	127	1

**Table 2:** *Y. enterocolitica* positive faecal culture positive patients (2008 to 2016)

	Date	Gender	Age	Serotype/biotype
Patient A	Oct-08	Female	40–50	Unknown (not O-3 & O-9)
Patient B	Oct-09	Male	10–20	O-3, unknown
Patient C	Apr-16	Female	60–70	O-47, 1a
Patient D	Aug-21	Female	30–40	O-8, unknown (ST188)
Patient E	Dec-23	Female	10–20	Unidentifiable, unknown (ST118)

## Discussion

Waterborne microbial diseases are a major public health concern, particularly in nations with poor sanitation [27]. Yersiniosis is well recognised as a potential waterborne disease. *Yersinia* species have minimal nutrient requirements, are highly adapted to aquatic environments and tolerate low temperatures, thereby surviving for long periods [28]. *Yersinia enterocolitica* has been reported in drinking water in many countries including Korea [29], Greece [28], Turkey [30], Canada [31], Germany [32], Norway [33, 34], Austria [35], Italy [36] and the USA [37]. None of these reports are from within the last 10 years however, and eight of them are over 30 years old. *Yersinia* species are typically susceptible to chlorine treatment of potable water, and much of the global water supplies are reliant on its use [38], but as water sources become more polluted, purification systems become less effective, and waterborne disease outbreaks have become commonplace [39]. Our recent study of coliforms detected in drinking water from private and public drinking water supplies across Ireland found three *Yersinia* species isolates with “good” identifications of *Yersinia enterocolitica* across a number of commercial identification systems [13]. The results of this study show that these results were unreliable and misleading, with two of the three strains confirmed to be *Yersinia proxima* isolates. Incorrect identification of *Yersinia* species by conventional laboratory instruments has been previously reported for *Yersinia hibernica* [40] and other *Yersinia* species [41]. This inadequacy may have profound implications for the investigation of food, water and clinical specimens for this important pathogen. It is clear that these systems need updating, in order to be able to correctly identify these and other newly described *Yersinia* species. Until then, next generation sequencing analysis appears to be the only dependable identification method, so laboratories that culture *Yersinia* species should have access to this technology either intrinsically or externally via a reference laboratory.

The reported incidence rate for *Yersinia enterocolitica* infection in Ireland (0.26 per 100,000 population in 2020 [15]) is below the European average (1.7 per 100,000) but similar to our nearest neighbour, the UK (0.2 per 100,000) [42]. The rate for the UK is deemed an underestimate due to the lack of routine screening; and culture (which lacks sensitivity) is the detection method of choice [43]. Faeces specimens are not routinely tested for *Yersinia enterocolitica* in our hospital, with just 1% of faeces specimens tested for this pathogen, using chromogenic selective agar. Just three cases of *Yersinia enterocolitica* infection have been diagnosed in our region in the last 10 years, representing an estimated aggregate crude annual incidence rate of 0.1 per 100,000 population for the period. When a hospital in Portsmouth in the South East of England began routinely testing faeces specimens for *Yersinia* by PCR in 2016, they detected 199 cases in the following 30 months, compared with just two cases in the preceding 30 months [44]. In that timeframe, this single laboratory diagnosed almost 60% of all *Yersinia* cases in England, and by extrapolating the findings of that laboratory, estimates of 7500 cases are undiagnosed annually in the UK [43]. Since the testing protocols in Ireland are also reliant on targeted culture methods, it can be assumed that there is also underreporting in this country.

The principle of “seek and you shall find” in the context of increasing the sensitivity of diagnostic tests is not a new one: across the spectrum of infectious gastrointestinal pathogens, molecular assays consistently outperform culture [45], and multiplex PCR systems have become the norm. In a report of *Campylobacter* infections in Ireland from 2004 to 2016 [46], there was a sharp increase observed in annual crude incidence (overall, in both sexes and in all age groups) at the time that diagnostic laboratories were transitioning from culture-based to molecular-based methods (2011). This transition to molecular detection of *Campylobacter* occurred in our laboratory in 2013, and the 5-year incidence showed a 10% increase after the change (942 Vs 1036 for 2008–2012 Vs 2014–2018). There were zero detections of *Shigella* from faeces in 2012 (prior to the introduction of molecular technology) compared with thirteen in 2014, and the 5-year incidence showed a 72% increase (39 Vs 67). The reverse effect was seen for *Salmonella* infections; however, the 5-year incidence showed a 10% decrease (143 Vs 118), but this decrease is most likely unrelated to test performance: The national incidence of *Salmonella* infections has been decreasing since 2000 on the back of intensive efforts to reduce *Salmonella* carriage and transmission at all stages of food production [47]. Comparison of toxigenic *E. coli* incidence is not possible because of definition changes at that time. In 2021, we expanded our quadruplex (*Salmonella*, *Shigella*, *Campylobacter* and Verotoxigenic *E. coli*) PCR system, to include targets for *Cryptosporidium* and *Giardia*: There was forthwith a three-fold increase in *Cryptosporidium* detections and a ten-fold increase in *Giardia* detections with the new system. In recent years, faecal gastrointestinal pathogen PCR tests have become increasingly broad in their scope and range of targets, and modern diagnostic laboratories need to embrace these technological advances. There is an abundance of molecular platforms available, see Supplementary Table 4 for a selection of thirteen kits and their manufacturer details.

## Conclusion

The evidence from our study is clear, and conventional identification systems are inadequate for the purpose of identifying *Yersinia* species. Species that are phenotypically indistinguishable from *Yersinia enterocolitica* are commonly misidentified, and determination of virulence, even when the identification is verified, poses significant challenges. There is no national reference laboratory in this country for *Yersinia* confirmation, despite a clear need for these microbiological capabilities either at a local or national level. The evidence from our closest neighbours (the UK) demonstrates likely under-reporting of *Yersinia* where culture-based methods are used, yet this remains the method of choice in this country. We recommend sentinel surveillance using modern molecular methods (of which many systems are available) for this organism, at least on a pilot basis. To quote Marcel Proust: “The only true voyage of discovery… would be not to visit strange lands but to possess other eyes”. We advocate for the use of fresh eyes to seek this significant pathogen.

### Supplementary Information

Below is the link to the electronic supplementary material.

Supplementary file1 (DOCX 284 KB)

## Data Availability

The authors confirm that all supporting data, code and protocols have been provided within the article or through supplementary data files.
