# Supplementary material for: Seek and you shall find: Yersinia enterocolitica in Ireland’s drinking water
Source: Ir J Med Sci. 2024 Feb 21;193(4):1885–90. doi: 10.1007/s11845-024-03641-5 (PMC11294261; doi:10.1007/s11845-024-03641-5)
Supplement: Supplementary file 1 — Supplementary file1 (DOCX 284 KB) [file 11845_2024_3641_MOESM1_ESM.docx]

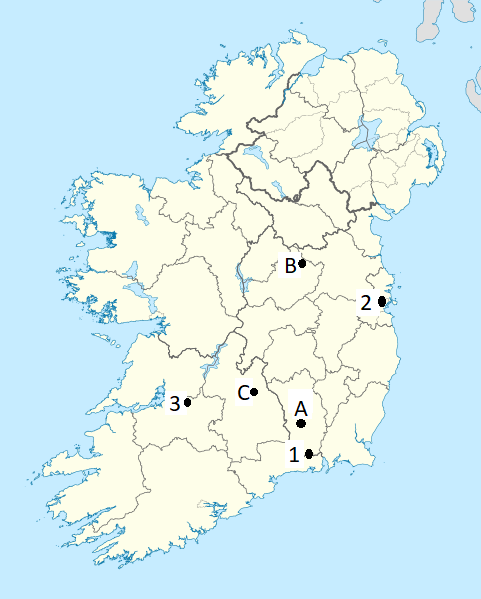


Supplementary Figure 1. Letters A to C: Water sampling sites; A) Thomastown, Co. Kilkenny. B) Castlepollard, Co Westmeath, C) Brittas, Co. Tipperary. Numbers 1 to 3: Public Health Laboratories; 1)University Hospital Waterford, 2) Lower Grand Canal St. Dublin, 3) HSE West Public Health Laboratory, Limerick.

| **TESTS** | **ACTIVE INGREDIENTS** | Isolate  A | Isolate  B | Isolate  C | *Yersinia enterocolytica*  NCTC10938 |
| --- | --- | --- | --- | --- | --- |
| ONPG | 2-nitrophenyl-ßD- galactopyranoside | POS | POS | POS | POS |
| ADH | L-arginine | NEG | NEG | NEG | NEG |
| LDC | L-lysine | NEG | NEG | NEG | NEG |
| ODC | L-ornithine | POS | POS | NEG | POS |
| CIT | Trisodium citrate | NEG | NEG | NEG | NEG |
| H2S | Sodium thiosulfate | NEG | NEG | NEG | NEG |
| URE | Urea | POS | POS | POS | POS |
| TDA | L-tryptophane | NEG | NEG | NEG | NEG |
| IND | L-tryptophane | POS | POS | POS | POS |
| VP | Sodium pyruvate | NEG | NEG | NEG | NEG |
| GEL | Gelatin | NEG | NEG | NEG | NEG |
| GLU | D-glucose | POS | POS | POS | POS |
| MAN | D-mannitol | POS | POS | POS | POS |
| INO | Inositol | POS | POS | POS | POS |
| SOR | D-sorbitol | POS | POS | POS | POS |
| RHA | L-rhamnose | NEG | NEG | NEG | NEG |
| SAC | D-sucrose | POS | POS | POS | POS |
| MEL | D-melibiose | NEG | NEG | NEG | NEG |
| AMY | Amygdalin | POS | POS | POS | POS |
| ARA | L-arabinose | POS | POS | POS | POS |
| OX | Oxidase | NEG | NEG | NEG | NEG |

Supplementary Table 1. API 20E results.

| **ACTIVE INGREDIENTS** | Isolate | Isolate | Isolate | *Yersinia enterocolytica* |
| --- | --- | --- | --- | --- |
|  | A | B | C | NCTC10938 |
| Lysine | Neg | Neg | Neg | Neg |
| Xylose | Pos | Pos | Pos | Pos |
| Phosphate | Neg | Neg | Neg | Neg |
| Maltose | Pos | Pos | Pos | Neg |
| Proline | Pos | Pos | Pos | Pos |
| Arabinose | Pos | Pos | Pos | Pos |
| γ-Glutamine | Pos | Pos | Pos | Pos |
| Malonate | Neg | Neg | Neg | Neg |
| Urea | Pos | Pos | Pos | Pos |
| 2-acetamido-2-deoxyglucoside & α-L-arabinoside | Pos | Pos | Pos | Pos |
| Trehalose | Pos | Pos | Pos | Pos |
| α-D-Glucoside | Neg | Neg | Neg | Neg |
| Fructose | Pos | Pos | Pos | Pos |
| Lysine | Neg | Neg | Neg | Neg |
| Arginine | Neg | Neg | Neg | Neg |
| Pyruvate | +/-^¥^ | Pos | Neg | Neg |
| Ornithine | Pos | Pos | Pos | Pos |
| Sucrose | Pos | Pos | Pos | Pos |
| Bis-Phosphate | Neg | Neg | Neg | Neg |
| Inositol | Pos | Pos | Pos | Pos |
| Esculin | Pos | Pos | Pos | Neg |
| Tryptophan deaminase | Neg | Neg | Neg | Neg |
| α-D-Galactoside | Neg | Neg | Neg | Neg |
| Citrate | Neg | Neg | Neg | Neg |
| Sorbitol | Pos | Pos | Pos | Pos |
| β-D-Glucuronide | Pos | Pos | Pos | Neg |
| Mannitol | Pos | Pos | Pos | Pos |
| β-D Galactoside | Neg | Neg | Neg | Neg |
| Arabitol | Pos | Pos | Pos | Neg |
| Raffinose | Neg | Neg | Neg | Neg |
| Cellobiose | Pos | Pos | Pos | Pos |
| Agmatine | Pos | Pos | Pos | Pos |

Supplementary Table 2. TREK Sensititre GNID results. ¥Tested in duplicate, differing results.

| Test | Mnemonic | Isolate | Isolate | Isolate | *Yersinia enterocolytica* |
| --- | --- | --- | --- | --- | --- |
|  |  | A | B | C | NCTC10938 |
| Ala-Phe-Pro-ARYLAMIDASE | APPA | NEG | NEG | NEG | NEG |
| ADONITOL | ADO | NEG | NEG | NEG | NEG |
| L-Pyrrolydonyl-ARYLAMIDASE | PyrA | POS | POS | POS | POS |
| L-ARABITOL | IARL | NEG | NEG | NEG | NEG |
| D-CELLOBIOSE | dCEL | POS | POS | POS | NEG |
| BETA-GALACTOSIDASE | BGAL | NEG | NEG | NEG | NEG |
| H2S PRODUCTION | H2S | NEG | NEG | NEG | NEG |
| BETA-N-ACETYL-GLUCOSAMINIDASE | BNAG | POS | POS | POS | NEG |
| Glutamyl Arylamidase pNA | AGLTp | NEG | NEG | NEG | NEG |
| D-GLUCOSE | dGLU | POS | POS | POS | POS |
| GAMMA-GLUTAMYL-TRANSFERASE | GGT | NEG | NEG | NEG | NEG |
| FERMENTATION/ GLUCOSE | OFF | POS | POS | POS | POS |
| BETA-GLUCOSIDASE | BGLU | POS | POS | POS | NEG |
| D-MALTOSE | dMAL | POS | NEG | POS | NEG |
| D-MANNITOL | dMAN | POS | POS | POS | POS |
| D-MANNOSE | dMNE | POS | POS | POS | POS |
| BETA-XYLOSIDASE | BXYL | NEG | NEG | NEG | NEG |
| BETA-Alanine arylamidase pNA | BAIap | NEG | NEG | NEG | NEG |
| L-Proline ARYLAMIDASE | ProA | NEG | NEG | POS | NEG |
| LIPASE | LIP | NEG | NEG | NEG | NEG |
| PALATINOSE | PLE | NEG | NEG | NEG | NEG |
| Tyrosine ARYLAMIDASE | TyrA | NEG | NEG | POS | NEG |
| UREASE | URE | POS | POS | POS | POS |
| D-SORBITOL | dSOR | POS | POS | POS | POS |
| SACCHAROSE/SUCROSE | SAC | POS | POS | POS | POS |
| D-TAGATOSE | dTAG | NEG | NEG | NEG | NEG |
| D-TREHALOSE | dTRE | POS | POS | POS | POS |
| CITRATE (SODIUM) | CIT | NEG | NEG | NEG | NEG |
| MALONATE | MNT | NEG | NEG | NEG | NEG |
| 5-KETO-D-GLUCONATE | 5KG | NEG | POS | POS | NEG |
| L-LACTATE alkalinization | ILATk | NEG | NEG | NEG | NEG |
| ALPHA-GLUCOSIDASE | AGLU | NEG | NEG | NEG | NEG |
| SUCCINATE alkalinization | SUCT | POS | POS | POS | NEG |
| Beta-N-ACETYL-GALACTOSAMINIDASE | NAGA | NEG | NEG | NEG | NEG |
| ALPHA-GALACTOSIDASE | AGAL | NEG | NEG | NEG | NEG |
| PHOSPHATASE | PHOS | NEG | NEG | NEG | NEG |
| Glycine ARYLAMIDASE | GlyA | NEG | NEG | NEG | NEG |
| ORNITHINE DECARBOXYLASE | ODC | NEG | POS | POS | POS |
| LYSINE DECARBOXYLASE | LDC | NEG | NEG | NEG | NEG |
| L-HISTIDINE assimilation | IHISa | NEG | NEG | NEG | NEG |
| COUMARATE | CMT | POS | POS | POS | POS |
| BETA-GLUCURONIDASE | BGUR | POS | POS | POS | NEG |
| O/129 RESISTANCE (comp.vibrio.) | O129R | POS | POS | POS | POS |
| Glu-Gly-Arg-ARYLAMIDASE | GGAA | NEG | NEG | NEG | NEG |
| L-MALATE assimilation | IMLTa | NEG | NEG | NEG | NEG |
| ELLMAN | ELLM | NEG | NEG | NEG | NEG |
| L-LACTATE assimilation | ILATa | NEG | NEG | NEG | NEG |

Supplementary Table 3. VITEK 2 GN Results.

| Product | Company | Location |
| --- | --- | --- |
| EntericBio® Dx | Serosep | Limerick, Ireland |
| BD MAX™ Extended Enteric Bacterial Panel | BD Molecular Diagnostics | New Jersey, USA |
| xTAG® gastrointestinal pathogen panel | Luminex | Texas, USA |
| BIOFIRE ® FILMARRAY ® Gastrointestinal (GI) Panel | Biomerieux | Marcy-l'Étoile, France |
| VERIGENE® Enteric Pathogens Test | Luminex | Texas, USA |
| Allplex™ GI-Bacteria(I) Assay | Seegene | Seoul, South Korea |
| Faecal Bacteria and Parasites 12-well | Ausdiagnostics | New South Wales, Australia |
| RIDA®GENE Bacterial Stool Panel | R-Biopharm | Darmstadt, Germany |
| LightMix® Modular Gastro Bacteria Multiplex | Roche | Basel, Switzerland |
| EasyScreen^TM^ Gastrointestinal Bacterial Targets | Genetic Signatures | New South Wales, Australia |
| QIAstat-Dx® Gastrointestinal Panel | QIAGEN | Hilden, Germany |
| Aeromonas + Y. enterocolitica RTPCR Kit | Viasure | Zaragoza, Spain |
| LiquidArray® Gastrointestinal | Bruker | Massachusetts, United States |

Supplementary Table 4 Gastrointestinal pathogen multiplex PCR kits capable of detecting *Yersinia enterocolitica*
